# Discovering SIFIs in Interbank Communities

**DOI:** 10.1371/journal.pone.0167781

**Published:** 2016-12-21

**Authors:** Nicolò Pecora, Pablo Rovira Kaltwasser, Alessandro Spelta

**Affiliations:** 1 Dept. of Economics and Social Sciences, Catholic University, Piacenza, Italy; 2 National Bank of Belgium, Brussels, Belgium; 3 Dept. of Economic and Business Sciences, University of Pavia, Pavia, Italy; College of Bioinformatics Science and Technology, CHINA

## Abstract

This paper proposes a new methodology based on non-negative matrix factorization to detect communities and to identify central nodes in a network as well as within communities. The method is specifically designed for directed weighted networks and, consequently, it has been applied to the interbank network derived from the e-MID interbank market. In an interbank network indeed links are directed, representing flows of funds between lenders and borrowers. Besides distinguishing between Systemically Important Borrowers and Lenders, the technique complements the detection of systemically important banks, revealing the community structure of the network, that proxies the most plausible areas of contagion of institutions’ distress.

## Introduction

The analysis of economic and financial networks, with the aim of measuring and monitoring the risks arising from Systemically Important Financial Institutions (SIFIs), has received a lot of attention [1] since a distress hitting these institutions could easily reverberate in the whole market [2].

In particular, the research in network theory has dedicated a huge effort to develop measures of interconnectedness, related to the detection of the most important player in a network, in order to capture the impact that an institution’s bilateral exposures have on other institutions within the system [3], [4], [5], [6], [7], [8].

In this article we argue that the identification of the modularity structure is of relevance in financial networks and complements the detection of systemically important banks, providing a measure of the most plausible areas of contagion of institutions’ distress. In presence of a community structure, indeed, an institution’s distress will not affect all the other components of the system homogeneously but, *in primis*, banks belonging to the same community.

On the other hand, not all banks in a community are equal, and some institutions might be special in the sense that they are linked to almost all others. These institutions could be seen potentially as SIFIs in the community they operate.

In general, centrality measures rank vertices according to their systemic importance without paying attention to whether the network is characterized by a community structure. On the contrary, several studies have analyzed the empirical characteristics of interbank networks in different jurisdictions [9], [10], finding the existence of a community structure [11], [12], [13], [14], [15], [16]. This topological characteristic indicates the presence of sets of institutions usually defined as very dense subgraphs, with few connections between them, as a result of preferential lending relationships at the micro-level [13], [17].

Despite the fact that centrality and community detection have been widely studied as independent phenomena from each other, to the best of our knowledge no unifying view of the two problems exists for directed networks. In this article, we try to fill this gap. We propose a new methodology to identify systemically important nodes and, simultaneously, the community structure of the network as well as the systemic importance of each node within communities.

Our method deals with relevant economic issues such as the determination of the systemic importance of each institution—both as a borrowers and/or as a lenders—in the whole network and in the community it belongs to, together with the identification of the areas of contagion for possible banks distress, provided by the detection of the community structure.

The method is based on non-negative matrix factorization (NMF). The NMF has been widely applied in the areas of data mining and machine learning since the initial work of Lee et al. [18]. It has been applied to a number of different areas such as pattern recognition [19], multimedia data analysis [20] and text mining [21]. Extensions of NMF have also been developed to accommodate various cost functions as needed in different data analysis problems, such as classification [22] and clustering [23]. Only recently NMF has been adapted to community detection. Zarei et al. [24] proposed a NMF-based algorithm for identifying fuzzy communities, Psorakis et al. [25] presented a community detection approach that employs a Bayesian NMF model to extract soft modules from networks. However, all of these NMF based methods only focus on the detection of communities, but none of them takes into account the identification of central nodes. The works by Shai et al. [26] and Li et al. [27] dealt with the possibility of overlapping structures in modular networks and how the modularity structure of a network is related to its resilience property. In fact, interconnected nodes play a key role in modular structures and their removal can have a deleterious effect on the network integrity, efficiency, and stability. Only recently, Cao et al. [28] have proposed a novel model to identify overlapping communities and central nodes in undirected network. Here we extend their methodology to directed graphs.

Since the technique we suggest is designed for directed networks, it has been applied to interbank networks. In an interbank network indeed, links are directed, representing flows of funds between lenders and borrowers. We test this method on the bilateral interbank exposures of the e-MID platform in order to find the community structure of the network during different periods and to evaluate the systemic importance of each bank within this market and within the communities the banks belong to. Data are taken from the Italian electronic broker market MID (Market for Interbank Deposits) run by e-MID S.p.A. Società Interbancaria per l’Automazione (SIA), Milan. The Italian electronic broker Market for Interbank Deposit (e-MID) covers the entire overnight deposit market in Italy.

In this context, it seems therefore appropriate to distinguish between Systemically Important Borrowers (SIBs) and Systemically Important Lenders (SILs). The risks for SILs lie on the asset side of its balance sheet, and will be transmitted to the rest of the system. On the contrary, banks borrowing large volumes distribute risks to the system through the liability side of the balance sheet.

We consider a weighted adjacency matrix as a mean of representing the interbank exposures network in which banks are connected (or adjacent) to other institutions. Using the NMF, such a matrix is approximated as the outer product of two lower dimensional matrices called borrowing and lending matrix respectively. Each element of these matrices represents the borrowing and lending systemic importance of each bank in each community.

In order to determine these matrices we exploits the connectivity information of the network highlighting the reinforcement relationships among nodes such that systemically important borrowers are pointed to by many systemically important lenders and systemically important lenders point to many systemically important borrowers [29]. This reinforcement relationship suggests that nodes that make themselves systemically important borrowers and lenders each other can be placed together in the same community [29] (see Materials and Methods for a formal definition).

Even if much of the focus within community detection methods has been put on identifying disjoint communities, it is well known that nodes in a network are naturally characterized by multiple community memberships [30], [31]. Our method takes into account this feature providing a soft membership distributions. Specifically, communities are retrieved independently from each other and vertices can belong to more than one community.

The soft partition solution gives us the opportunity to connect two fundamental features of interbank networks: the existence of communities and the core-periphery structure [14], [17]. While communities are often thought of as densely linked clusters of nodes, the core-periphery structure represents a network composed of a sparsely connected periphery and a densely connected core [32], [33], [34] that lacks internal communities. However, within our model, overlapping communities lead to a global core-periphery network structure, where the dense network core is formed as a result of many overlapping communities.

Finally we also provide a hard partition scheme, where overlapping communities are not allowed by assigning nodes to the community in which they have the highest degree of membership.

## Materials and Methods

Let *G* = (*V*, *E*) be a directed and weighted graph representing financial transactions taking place in the interbank market, where *V* is the *n*-dimensional set of banks and *E* the *m*-dimensional set of financial transactions. Graphically, banks are represented by nodes and transactions by edges. Let **W**_*i*,*j*_ be the amount that bank *j* lends to bank *i* in a certain period. The collection of all the interbank transactions between financial institutions during the same period leads to the matrix of exposures **W**_*n*×*n*_, where **W**_*i*,*j*_ > 0 if a transaction between *i* and *j* takes place while **W**_*i*,*j*_ = 0 otherwise. We call this matrix the *weighted-adjacency transaction* matrix. Let K∈N be the maximum number of communities in the network at a certain time. In empirical works though, *K* needs to be fixed on the basis of the desired level of detail: a low number of components only yields the strongest structures, whereas using a high number of components faces the risk of overfitting noise. In the extreme case of *K* = 1, the borrowing and lending scores are computed for the whole network structure, without assessing the presence of a community structure inside the network. In what follows we assume *K* is known a priori (we will relax this assumption in showing the application of the methodology).

The NMF method consists in factorizing the exposures matrix **W** into two matrices, **B** and **L**, such that both matrices have no negative elements, i.e. $\text{B}\in R_{+}^{n\times K}$ and $\text{L}\in R_{+}^{K\times n}$. The element **B**_*ik*_ corresponds to the borrowing systemic importance of bank *i* within community *k*. By analogy, the element **L**_*ki*_ represents the systemic importance of bank *i* within the community *k* in terms of its lending activity. It is straightforward to interpret **B**_*ik*_ **L**_*kj*_ as the contribution, in terms of model fitting, of the *k*-th community to the edge **W**_*ij*_. In other words, the interaction ${\hat{\text{W}}}_{ij}=\sum_{k=1}^{K}{\hat{\text{W}}}_{ij}^{k}=\sum_{k=1}^{K}$ **B**_*ik*_ **L**_*kj*_ between nodes *i* and *j* is the result of the sum of their participation in the same communities [25], [35]. Therefore, $\hat{\text{W}}$ is a summation of *K* rank-1 matrices and each ${\hat{\text{W}}}_{ij}^{k}$ denotes the number of pairwise interactions in the context of community *k*. Thus $\hat{\text{W}}$ is an approximation of the original matrix **W** and the model fit can be easily calculated as $100(1-\frac{\sum \sum {\left(\text{W}-\hat{\text{W}}\right)}^{2}}{\sum \sum {\left(\text{W}\right)}^{2}})$.

We call the sum over each column of the matrix **B** and over each row of **L** as **s**^*B*^ = ∑_*k*_ **B**_*ik*_ and **s**^*L*^ = ∑_*k*_ **L**_*kj*_ respectively. If each column of **B**_:,*k*_ and each row of **L**_*k*,:_ is normalized to one, dividing it by ${\text{s}}_{1,k}^{B}$ and ${\text{s}}_{k,1}^{L},$ the elements **B**_*ik*_ and **L**_*ki*_ can be seen as the proportion of borrowing and lending systemic importance of bank *i* into community *k* since now ∑_*k*_ **B**_*ik*_ = 1 and ∑_*k*_ **L**_*ki*_ = 1.

Since we are dealing with overlapping communities, a soft partition scheme is proposed by assigning to each node the percentage of its strength centrality that belong to that community $a_{i,k}=\frac{\sum_{j=1}^{n}\left({\hat{\text{W}}}_{ij}^{k}+{\hat{\text{W}}}_{ji}^{k}\right)}{\sum_{k=1}^{K}\left[\sum_{j=1}^{n}\left({\hat{\text{W}}}_{ij}^{k}+{\hat{\text{W}}}_{ji}^{k}\right)\right]}.$

Such an edge decomposition can then be used also to assign nodes to communities according to a hard partition scheme, assigning each bank to the community in which it has the highest impact in terms of strength.

In order to compute **B** and **L**, we consider the following minimization problem

$$\begin{array}{c} \min_{\text{B}\in R_{+}^{n\times K},\text{L}\in R_{+}^{K\times n},}\frac{1}{2}∥\text{W}-\text{BL}{∥}_{F}^{2} \end{array}$$ (1)

where $∥•{∥}_{F}^{2}$ is the Frobenius norm. The optimization problem results in

$$\begin{array}{ccc} \text{B} & \geq & 0,\\ \text{L} & \geq & 0 \end{array}$$ (2)

$$\begin{array}{ccc} \nabla_{\text{B}} & = & {\text{BLL}}^{T}-{\text{WL}}^{T}\geq 0,\\ \nabla_{\text{L}} & = & {\text{B}}^{T}\text{BL}-{\text{B}}^{T}\text{W}\geq 0 \end{array}$$ (3)

$$\begin{array}{ccc} \text{B}⊛({\text{BLL}}^{T}-{\text{WL}}^{T}) & = & 0,\\ \text{L}⊛({\text{B}}^{T}\text{BL}-{\text{B}}^{T}\text{W}) & = & 0 \end{array}$$ (4)

where ⊛ is the Hadamard product and Eqs (3) and (4) are the Karush-Kuhn-Tucker conditions.

We can solve this problem using the gradient descendent method [36] by choosing a set of initial values for **B** and **L**.

$$\begin{array}{c} {\text{B}}_{ik}\leftarrow {\text{B}}_{ik}\frac{{\left({\text{WL}}^{T}\right)}_{ik}}{{\left({\text{BLL}}^{T}\right)}_{ik}} \end{array}$$ (5)

$$\begin{array}{c} {\text{L}}_{jk}\leftarrow {\text{L}}_{jk}\frac{{\left({\text{B}}^{T}\text{W}\right)}_{jk}}{{\left(\text{B}{\text{B}}^{\text{T}}\text{L}\right)}_{jk}} \end{array}$$ (6)

The expressions in Eqs (5) and (6) represent the borrowing and the lending score of banks *i* and *j* in community *k* respectively. For example, the borrowing score **B**_*ik*_, which measures the capability of bank *i* to borrow from banks belonging to community *k*, is obtained by multiplying the *i*-th row of matrix **W** (which collect flows borrowed by bank *i*) with the *k*-th column of matrix **L** (which collect the lending score of each bank in community *k*). A similar argument applies to **L**_*jk*_.

Once matrices **L** and **B** are obtained, we can calculate the weighted-adjacency transaction matrix approximation ${\hat{\text{W}}}^{k}$ belonging to each community and then we can assign nodes to communities depending on the normalized degree that each bank has in each community.

It is worth to notice that since Problem (1) is an unconstrained problem, the order of magnitudes of the lending score is 4 times the one of the borrowing score. Despite this fact, the ranking position of the nodes in the two indexes are not affected by this issue because the NMF is scale invariant. One can multiply **B** by some constant *c* and **L** by 1/*c* to obtain different **B** and **L** without changing their product. Then, we can not say whether a bank is more systemically important as a lender or as a borrower, but we can rank banks in terms of borrowing and lending importance separately. In other words, we can only look at the importance of each bank inside one of these two sets (borrowing and lending) but we can not compare them.

## Results

In this section we present the application of our method to the e-MID dataset. We consider a set of 354 banks, each of them is represented by a node of the interbank network. The links between banks represent the amount of their exposures vis-a-vis the rest of the reporting banks, measured on a monthly basis from the beginning of 1999 to the end of 2012.

Let us consider first the Borrowing and Lending scores obtained disregarding the presence of a community structure, namely setting the number of communities equal to one. Notice that this procedure leads to the same results obtained by the HITS algorithm [29] (see S1 File). Similarly to a feedback centrality measure, the ranking of a bank is calculated taking into account the exposures of its neighbors, and the neighbors’ centrality scores in turn, will be calculated taking into account the exposures of the neighbors of the neighbors, etc.

**Fig 1:**
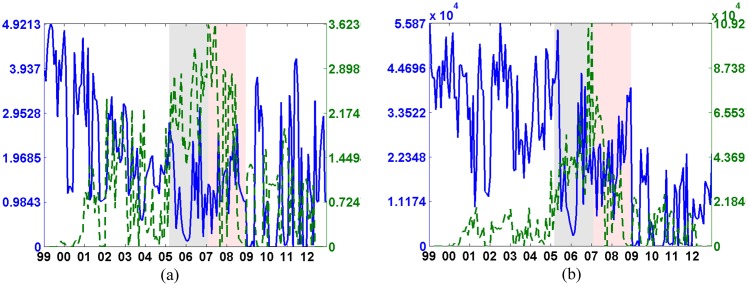
Time evolution of the borrowing (a) and lending (b) score for rank-1 NMF from 1999 to 2012 at a monthly frequency. The solid blue line refers to the sum of Italian banks’ scores while the dashed green line is associated to the sum of foreign banks’ scores. The gray shaded area emphasizes the pre-crisis period (2005-Q1:2007-Q1) while the red area indicates the crisis period (2007Q1:2008-Q4). The x-axis in both subplots refers to years while the left y-labels report the sum of the borrowing (a) and lending (b) scores for Italian banks, the right y-labels are associated to magnitude of the score of the foreign banks.

Fig 1 presents the dynamics of the borrowing (a) and of the lending (b) scores. For each measure we aggregate the scores associated to Italian (solid blue line) and to foreign banks (dashed green line). The course of the two scores indicates that the sum of the systemic importance associated with Italian banks decreased during the recent financial crisis while the opposite happened for foreign institutions. Moreover while Italian banks’ borrowing scores approximately turn back to the pre-crisis level after 2009, the lending score settles down to lower values. The scores of foreign banks peak at the beginning of the crisis: the borrowing score starts rising from 2005 and it keeps increasing until 2007 whereas the lending score has a steep build up from 2006, collapsing after 2007.

These dynamics underline different economic trends. During the years 1999-2005 foreign financial institutions joined the e-MID interbank market, borrowing mostly from Italian banks. During the pre-crisis period (gray background) this trend grew up, but the most systemically important lenders turned out to be other foreign financial institutions. The dynamics reverted when the crisis unfolded (red background): foreign banks suddenly stopped to lend to other institutions, and smoothly decreased their borrowing operations. Italian banks, on the contrary, increased their lending activities.

**Fig 2:**
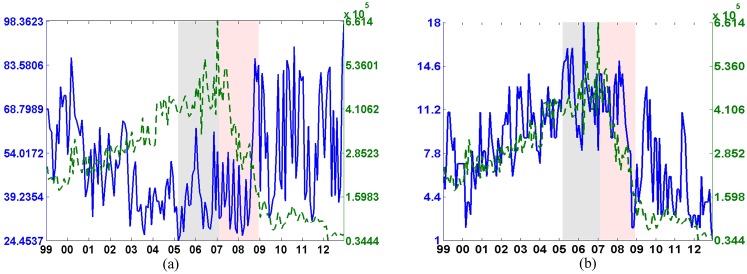
Traded volume (a) and number of communities (b). Time evolution of the model fit (blue line) versus the traded volume (green dashed line) is shown in the panel (a). Panel (b) displays the number of communities (blue line) versus the traded volume (dashed green line). The x-axis displays the time evolution. The y-right axis displays the traded volume in millions of dollars while on the y-left axis we report the model fitting (left panel), and the number of communities (right panel).

Despite the centrality measures help the understanding of the relative position (the systemic importance) of each bank during different time periods, the model fit widely oscillates from 24 to 95%, and it also displays a negative correlation with the traded volume during the whole sample, as reported by Fig 2(a). The rank-1 NMF decomposition is well suited to describe the borrowing and lending relationships only at the beginning of the time sample, from 1999 to 2002, or after 2008, when the transaction volume lowered. However, it leaves out substantial topological information while computing the systemic importance of financial institutions during the market euphoria and the subsequent crash, namely from 2003 to 2008.

This opens the issue of whether a better micro investigation, at a community level, can enhance the understanding of the systemic importance associated to each financial institution. Thus we investigate the clusterization trend that affected the interbank market during the last decade along with the centrality scores of institutions within each community. To do so, we adopt a heuristic approach fixing at 90% the data variability we want to replicate, and looking for the number of communities that can jointly meet this goodness of fit (see also S1 File).

Fig 2(b) shows the evolution of the number of communities (solid blue line) and the traded volume (dashed green line) over time. The positive correlation between the two quantities clearly appears until the end of the crisis. The growing traded volume is positively related with the increasing number of communities of the interbank market before the 2007 collapse. When most of the banks interrupted transactions, the number of communities decreased as well. It is worth mentioning that after 2009 the modules widely oscillated even if the overall traded volume remains low.

The link between the number of communities and the traded volume is helpful in understanding the interbank market dynamics. Banks are repeatedly engaged in transactions with other banks within the same community, while transactions between banks of different communities are much lower. Several factors can explain why banks form modules in the interbank network. It is well known that information asymmetries, moral hazard, adverse selection and market frictions influence the behavior of banks in the interbank network. Moreover, differently from other studies [12, 15, 37, 38], we provide evidence that the e-MID interbank network, although being characterized by communities, does not display a persistent structure over time.

Additionally, since this technique admits an overlapping or soft-partitioning solution, i.e. communities are allowed to share members, it seems natural to investigate the soft-membership distributions of each bank across time, which quantify how strongly each individual participates in each group. In other words we can explore the degree of fuzziness in the network by collecting, for each time and for each bank, the coefficient of variation of the degree membership across communities. The coefficient is defined as the ratio of the standard deviation of the degree membership to the mean

$$\begin{array}{c} V_{i,t}^{B}=\frac{{\left(\frac{1}{K_{t}}\sum_{k=1}^{K_{t}}{\left({\text{a}}_{i,k}^{t}-{\bar{\text{a}}}_{i}^{t}\right)}^{2}\right)}^{\frac{1}{2}}}{{\bar{\text{a}}}_{i}^{t}} \end{array}$$

where *K*_*t*_ is the number of communities at time *t*, ${\text{a}}_{i,k}^{t}$ is the degree of membership of the *i*-th nodes in the *k*-th community at *t* and ${\bar{\text{a}}}_{i}^{t}$ is the average membership degree for the *i*-th nodes across communities in which it participates at *t*,

$$\begin{array}{c} {\bar{\text{a}}}_{i}^{t}=\frac{1}{K_{t}}\sum_{k=1}^{K_{t}}{\text{a}}_{i,k}^{t} \end{array}$$

The same index is also applied to the borrowing and lending scores. A financial institution that presents a low coefficient of variation, having a membership distribution that is closer to uniform, belongs to different communities. On the contrary, a high coefficient of variation, means that a bank, having a unimodal membership distribution, belongs only to the corresponding community.

**Fig 3:**
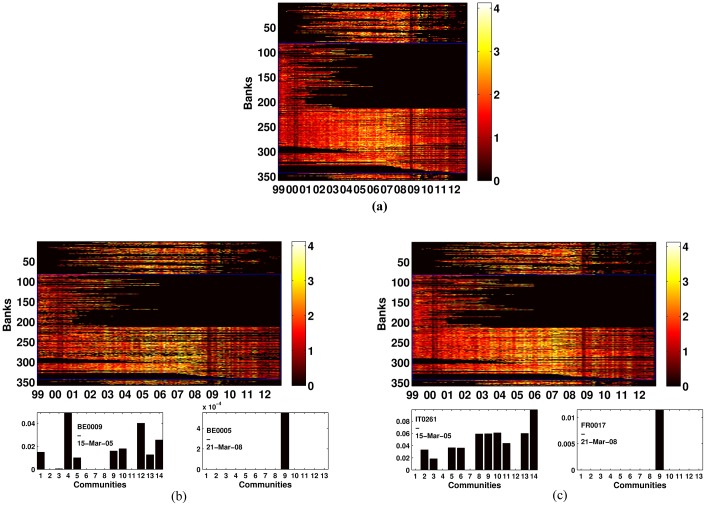
Time evolution of the coefficient of variation. Time evolution of the coefficient of variation of the membership distribution (a) and for the borrowing (b) and the lending (c) scores. We distinguish between Italian and foreign banks behavior encapsulating Italian banks into a blue rectangle. In each subplot the y-left axis shows the number of anonymous banks operating while the x-axis denotes years. The color bars emphasize the coefficient of variation value. The bottom panels show an example of the degree membership distribution associated with banks in particular periods: it can be dispersed across communities or fully concentrated into a particular module.

Fig 3 displays the coefficient of variation of degree of membership of each node (a), of the borrowing (b) and of the lending (c) scores across communities. The coefficient displays approximately the same pattern for all indexes, signaling an increase in the variability during the pre-crisis and crisis years. Therefore institutions, during the pre-crisis and crisis time, increased operations inside each community, without (or with small) overlapping as reported by the high coefficient of variation of the partition scheme. On the contrary, in non-crisis periods, not only the number of communities is lower than during crisis period (see Fig 2), but banks participate in different clusters as a borrower or lender. Fig 3 also indicates that half of the Italian banks were active for the whole sample size while other stopped to exchange funds during the first years (black area). On the contrary foreign banks were particularly active during the years of the financial crisis. Moreover, together with the growth in the number of communities, the e-MID interbank market was affected by a strong split of banks within each community during the crisis phase. As an example the small bottom plots of Fig 3 show the soft-membership distribution across communities for specific banks in different time periods.

Finally, in order to give a simple overview of the results obtained by the application of NMF to the e-MID dataset, we show the network community structure and the relative scores for banks in September 2008 when Lehman Brothers bankruptcy occurred.

**Fig 4:**
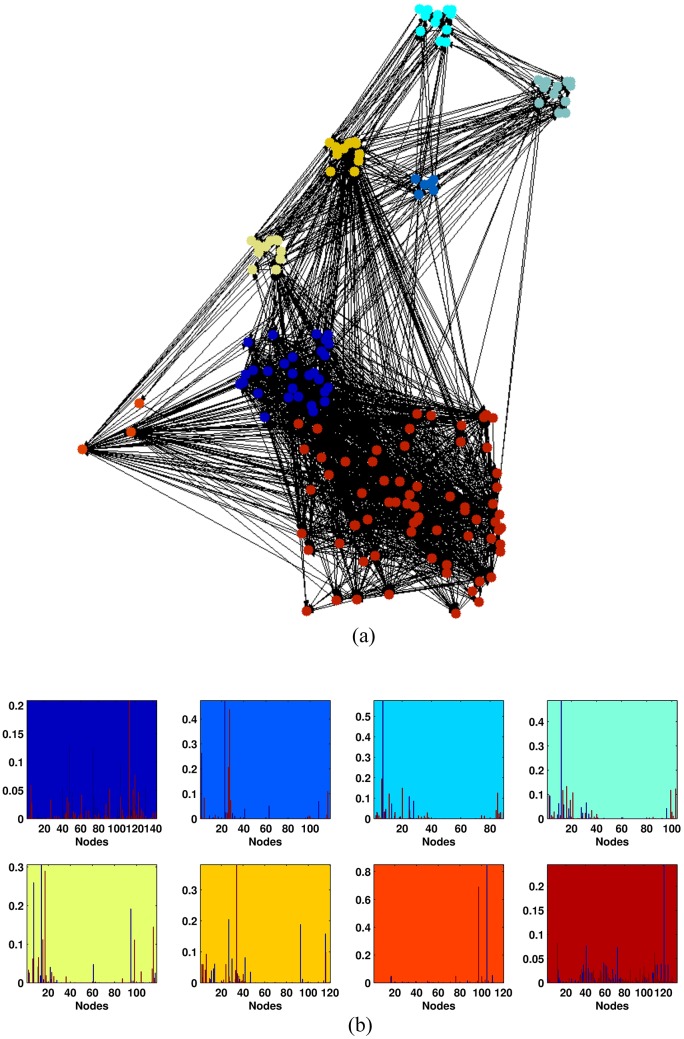
The network community structure of the e-Mid interbank market. The network community structure of the e-Mid interbank market during September 2008 (a) together with the borrowing (blue bars) and lending (red bars) scores for each community (b). The communities are emphasized with different background colors. On the x-axis we display the number of nodes, on the y-axis the strength of the scores. While we use a hard partition scheme in order to visualize the network, the scores are calculated using a soft partition scheme.

The interbank network displays eight communities, emphasized using different colors. Fig 4(a) shows a network representation of the relationships among banks, using a hard partition scheme, where nodes are assigned to the community that mostly contributed to their scores. On Fig 4(b) we show the borrowing (blue bar) and lending (red bar) scores for each bank inside each community. The background colors represent communities the banks belong to. Differently from the network visualization, the nodes are associated with each community via a soft partition scheme, therefore, a single bank can belong to different communities. The scores indicate that few banks operate as SIBs or SILs within each community.

### NMF method vs weighted degree measures: a comparison with Basel III

We compare the ranking obtained using the NMF method with the ones obtained with the methodology proposed by Basel III (in- and out-weighted degrees). The weighted in-degree and the weighted out-degree, which measure the total amount of borrowing (ingoing) and lending (outgoing) respectively are formally defined as:

$$\begin{array}{c} k_{i,in}^{w}=\underset{j=1}{\sum^{n}}{\text{W}}_{i,j}\;\;k_{i,out}^{w}=\underset{i=1}{\sum^{n}}{\text{W}}_{i,j} \end{array}$$

Specifically, we ask the following question: “how many of the SIFIs that we identify with our methodology are also picked out by the approach employed by the Basel Committee?” Basel III applies a bucketing approach with a certain cutoff point and labels as Systemically Important banks those that lie above the threshold. According to the Basel Committee on Banking Supervision, 28 banks were classified as Globally-Systemically Important in November 2012. We adopt a similar bucketing approach and label banks as systemically important if their ranking falls within the upper 20-th percentile of importance.

**Fig 5:**
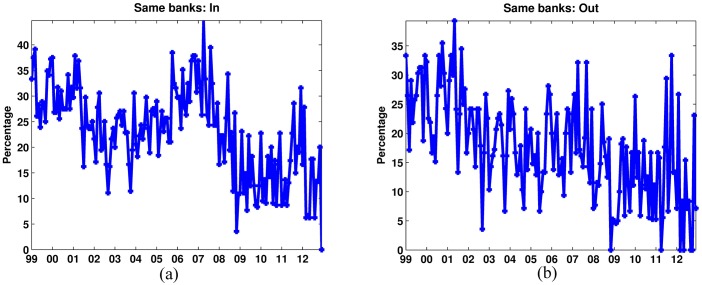
NMF method vs weighted degree measures. Panel (a) shows the percentage of banks identified as SIBs (SIFIs for the borrowing component) by both methods. Panel (b) shows the percentage of banks identified as SILs (SIFIs for the lending component) by both methods.

The results are shown in Fig 5(a) and 5(b). The figure displays the percentage of banks that were labeled as SIFIs within our method and within the Basel III technique simultaneously. The difference between the two methods is considerably large. In the best of the cases, there is an approximate 40%-50% coincidence among the banks identified by the borrowing and lending scores and the Basel III method. The percentage of coincidence then reaches zero toward the end of the sample.

The difference in the rankings produced by the two measures relies on the fact that the weighted degree measure provides no information about the higher order exposures of banks, i.e. no information is provided about the way in which bilateral risks compound each other affecting the overall system. At the same time, from this measure, it is not clear how the network structure and the fragility of the system feed-back into the individual banks. On the other hand, being a feedback measure of systemic importance, the NMF evaluates not only the individual exposures, as the current Basel III framework does, but the algorithm is also able to capture the risks that individual institutions place into the system. Furthermore, and at the same time, it also takes into account how the exposures at the system-wide level affect the individual institutions. Hence, this ranking methodology considers both the micro and the macro dimensions of banks’ exposures in the interbank market and emphasizes the importance of higher order interconnections to identify SIFIs.

### Evaluating the algorithm

In this section we compare our algorithm with several well-known community detection methods. Since our method produces both soft and hard partition schemes, we compare the goodness of the communities obtained by both solutions against methods that produce crisp assignment (non-fuzzy) or fuzzy assignment. With crisp assignment, the relationship between a node and a cluster is binary. That is, a node *i* either belongs to cluster *c* or does not. With fuzzy assignment, each node is associated with communities in proportion to a belonging factor. Thus we compare our hard partition solution against methods that produce crisp assignment and the soft partition solution against methods that produce fuzzy assignment.

In particular we consider the modularity maximization method [39], [40], the Louvain method [41] and the K-means algorithm [42] for crisp assignment; the C-means algorithm [43], [44], the Clique Percolation [45], the Lancichinetti et al. [46] and Huang et al. [47] methods for fuzzy assignment.

Since our algorithm is applied to networks for which the communities are not known in advance, we need a measure to quantify the goodness of the communities detected by each technique. In other word, we would like to know which of the divisions produced by the different algorithms is the best for the given network. To answer this question, for each time period, we define two modularity measures that show the quality of a particular division of a network. These two measures are the crisp and the fuzzy modularity for directed weighted network, defined as:

$$\begin{array}{c} Q^{C}=\frac{1}{m}\sum_{i,j}\left[{\text{W}}_{ij}-\frac{{\text{s}}_{i}^{in}{\text{s}}_{j}^{out}}{m}\right]\delta \left(c_{i},c_{j}\right) \end{array}$$

$$\begin{array}{c} Q^{F}=\frac{1}{m}\sum_{c}\sum_{i,j}\left[{\text{W}}_{ij}-\frac{{\text{s}}_{i}^{in}{\text{s}}_{j}^{out}}{m}\right]{\text{a}}_{ic}{\text{a}}_{jc} \end{array}$$

respectively, where **s**^*in*^ and **s**^*out*^ are the in- and out-strength respectively, $m=\sum_{i}{\text{s}}_{i}^{in}=\sum_{j}{\text{s}}_{j}^{out}$. The difference between the two measures relies on the last term: *δ*(*c*_*i*_, *c*_*j*_) is the Kronecker delta symbol, and *c*_*i*_ (*c*_*j*_) is the label of the community to which node *i* (*j*) is assigned; **a**_*ic*_ (**a**_*jc*_) is the degree of membership of node *i* (*j*) in the community *c*.

**Fig 6:**
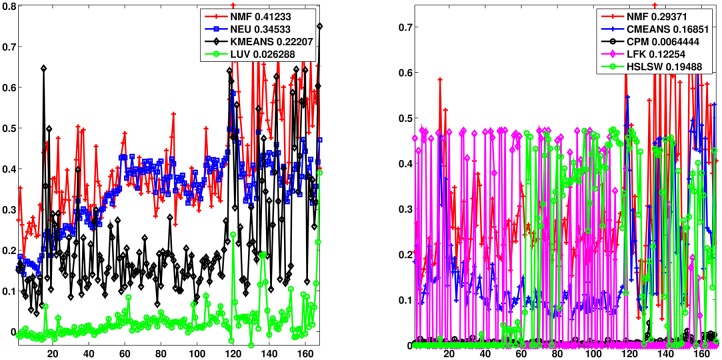
The modularity comparison with different algorithms. The modularity of different algorithms for hard (a) and soft partition solutions (b) along time. Beside the names of each algorithm we report the average of the modularity values over the entire data sample. In the legends, NMF refers to our algorithm, Von Newman (NEW), K-means (KMEANS) and Louvain method (LUV) for hard partition solution. The soft partition solution of our method (NMF) is also compared with the C-means algorithm (CMEANS), with the Clique Percolation Method (CPM) and with the algorithms of Lancichinetti et al. (LFW) and Huang et al. (HSLSW).

Fig 6 reports the results for the different methods. We calculate the modularity metrics for each period in the data sample and in the legend, near the name of each algorithm, we report the average modularity value. Fig 6(a) encompasses the results about the hard partition solutions, while in the Fig 6(b) we show the modularity for the soft partition solutions. In both cases, on average, our method outperforms the other algorithms even if in some period the other techniques provide a higher modularity. Moreover comparing the soft and the hard partition solutions of our method, one can notice that in the middle of the data sample, when the number of community increases, with banks operating in different communities with low overlapping, the modularity of the hard partition solution becomes higher than the one obtained with a soft partition solution.

### Community and Core-Periphery structure

Our technique based on NMF helps in gaining well-founded insights into interbank networks. In particular, this subsection is devoted to show that the existence of communities and of a core-periphery structure [17], [14] are not two mutually exclusive features. Core-periphery structure captures the notion that many interbank networks decompose into a densely connected core and a sparsely connected periphery [32], [33], [34].

Usually the core lacks internal communities, however, within our model, overlapping communities lead to a global core-periphery network structure, where the dense network core is formed as a result of many overlapping communities.

**Fig 7:**
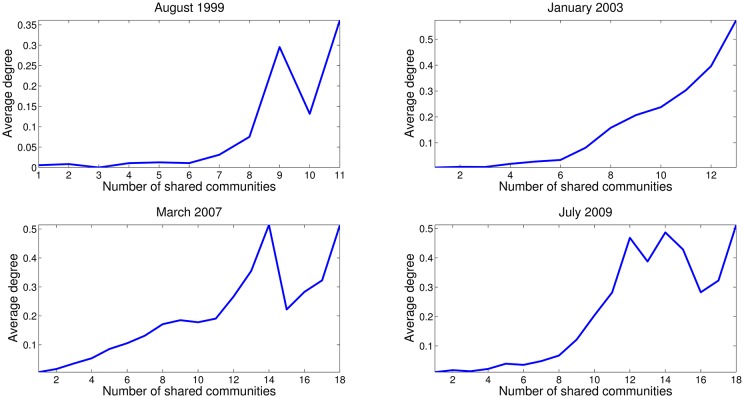
Average degree of nodes that share a given number of communities. The average degree is increasing in the number of shared community. This means that nodes that belong to overlapping communities are more densely connected that non overlapping parts of communities.

Fig 7 shows the average degree for nodes that share a given number of communities. Results suggests that the average degree is increasing in the number of shared communities meaning that community overlapping is more densely connected that non overlapping parts of communities.

## Discussion

In this article, we proposed a new methodology to identify central nodes and, simultaneously, to detect the community structure in directed graphs. A high centrality is associated with a high systemic importance of a bank and the network community structure helps to identify the most probable areas of contagion of a bank’s distress. SIFIs are identified according to two indicator-based measures that we name borrowing score and lending score. In so doing we are able to distinguish between risks arising from exposures on the asset and on the liability side of banks’ balance sheet. In other words we discriminate banks as Systemically Important Borrowers or Systemically Important Lenders as suggested by the reform proposed by Basel III.

Our work reveals that the systemic importance associated with Italian banks decreased during the recent financial crisis while the opposite happened for foreign institutions. Since interbank market displays the existence of a community structure, our method outperforms, in term of goodness of fit, the centrality scores calculated along with a rank-1 factorization. Indeed, the borrowing and lending scores, calculated without assessing the presence of a community structure, although being informative about some market dynamics, fail in recovering the market euphoria and the subsequent crash of the recent past. In fact, as the transaction volume increased, the number of communities into the market rose as well. On the contrary, during the burst phase, when most of the banks interrupted transactions, also the number of communities decreased on average.

We also investigated whether the increase in the number of communities is associated with a stronger partition of the financial institutions within each community or whether banks operate across different communities. Results indicate a different behavior affecting financial institutions in normal time or in periods of distress. Together with the growth in the number of communities, the e-MID interbank market was affected by a strong split of banks within each community during the recent financial crisis with few banks operating as SIBs or SILs.

## Supporting Information

S1 File (PDF)

S1 Fig Model fit comparison. Model fit comparison for rank-1 approximation of the original network (blue line) and for the network sample created by the null model (red line). While the original dataset shows a “V” shaped model fit, the fit produced by the null model seems not to be affected by the traded volume changes over time.
(TIF)

S2 Fig Summary statistics of the borrowing/lending scores. Panel (a) shows the statistics computed over time for the borrowing score, showing in each period what is the mean value of the borrowing score and its standard deviation. Panel (b) shows the same statistics for the lending scores. The bottom subplots show the statistics computed over the number of banks. Panel (c) shows, for each bank the mean value of the borrowing score and its standard deviation. Panel (d) encompasses the same statistics for the lending score.
(TIF)

S1 Table Top 10 periods and banks with the highest borrowing and lending scores. (TIF)

S2 Table Top 10 periods and banks with the highest borrowing and lending scores mean. (TIF)

S3 Table Top 10 periods and banks with the highest borrowing and lending scores standard deviation. (TIF)

S3 Fig Model convergence. Relative error as a function of the number of iteration for all the time periods under analysis.
(TIF)

S4 Table Descriptive statistics. (TIF)

S4 Fig Italian vs Foreign Banks dynamics. (TIF)
