# Correction: Discovering SIFIs in Interbank Communities

**DOI:** 10.1371/journal.pone.0176542

**Published:** 2017-04-20

**Authors:** Nicolò Pecora, Pablo Rovira Kaltwasser, Alessandro Spelta

The second affiliation for author Pablo Rovira Kaltwasser is not indicated. Pablo Rovira Kaltwasser is also affiliated with: Dept. of Economic and Business, University of Leuven, Leuven, Belgium.
