# Supplementary material for: Discovering SIFIs in Interbank Communities
Source: PLoS One. 2016 Dec 21;11(12):e0167781. doi: 10.1371/journal.pone.0167781 (PMC5176285; doi:10.1371/journal.pone.0167781)
Supplement: S1 File — (PDF) [file pone.0167781.s001.pdf]

Discovering SIFIs in interbank communities

Nicolò Pecora[1], Pablo Rovira Kaltwasser[2,3], Alessandro Spelta[4,5,*]

1 Dept. of Economics and Social Sciences, Catholic University, Piacenza, Italy
2 Dept. of Economics, Catholic University, Leuven, Belgium
3 National Bank of Belgium
4 Dept. of Economics and Finance, Catholic University, Milano, Italy
5 Complexity Lab in Economics, Milano, Italy

*** alessandro.spelta@unicatt.it**

Supporting Information

Relationship between NMF and HITS algorithm

The HITS algorithm [1] was originally intended to discover the most central pages for broad search topics in the context of the WWW. It uses appropriate eigenvectors (or singular vectors) decomposition to compute the authority ($\mathbf{a}_i$) and the hubness ($\mathbf{h}_i$) of node i. Authority measures prestige: nodes who many other nodes point to are called authorities. If a node has a high number of nodes pointing to it, it has a high authority value and this quantifies its role as a source of information. On the contrary, a hub is an actor referring to many authorities and its score measures acquaintance. Essentially, a good hub points to many good authorities and a good authority is pointed to by many good hubs.

If we denote the weighed-adjacency transaction matrix as $\mathbf{W}$, the HITS algorithm leads to hubs and authorities computed iteratively

$$\mathbf{h}^{(t+1)} = \mathbf{W}\mathbf{a}^{(t)} \tag{1}$$

$$\mathbf{a}^{(t+1)} = \mathbf{W}^T\mathbf{h}^{(t+1)} \tag{2}$$

where vectors $\mathbf{a} = [a_1, ..., a_n]^T$ and $\mathbf{h} = [h_1, ..., h_n]^T$ yield respectively the authority and the hub scores of all nodes. Performing power iteration method on $\mathbf{W}\mathbf{W}^T$ and $\mathbf{W}^T\mathbf{W}$, $\mathbf{a}^{(t)}$ and $\mathbf{h}^{(t)}$ will converge respectively to the principal eigenvectors $\mathbf{a}$ and $\mathbf{h}$ of the symmetric semi-positive definite matrices $\mathbf{W}\mathbf{W}^T$ and $\mathbf{W}^T\mathbf{W}$.

Given the $n \times n$ non-negative matrix $\mathbf{W}$ (i.e. $\mathbf{W}_{ij} \geq 0$) and a reduced rank $K = 1$ approximation, the NMF rank-one decompositions is equivalent to a SVD-decomposition problem of finding two non-negative vectors $\mathbf{a} \in \mathbb{R}_+^{n\times 1}$ and $\mathbf{h} \in \mathbb{R}_+^{n\times 1}$ together with a scalar $d \in \mathbb{R}_+$ that approximate $\mathbf{W}$, i.e.

$$\mathbf{W} \approx \mathbf{a}d\mathbf{h}^T = \hat{\mathbf{W}}$$

We need to prove that a and h are local minimizers of the function $\frac{1}{2} \parallel \mathbf{W} - \hat{\mathbf{W}} \parallel_F^2$, i.e. we have to solve the following optimization problem

$$\min_{\mathbf{a}\in\mathbb{R}_+^{n\times 1}\mathbf{h}\in\mathbb{R}_+^{n\times 1}, d\in\mathbb{R}} \frac{1}{2} \parallel \mathbf{W} - \mathbf{a}d\mathbf{h}^T \parallel_F^2 \tag{3}$$

where $\| \bullet \|_F^2$ represents the Frobenius norm. Equivalently $\hat{\mathbf{W}}$ can be written as

$$\begin{aligned} \hat{\mathbf{W}} &= \mathbf{a} d \mathbf{h}^T = \mathbf{a} d^{1/2} d^{1/2} \mathbf{h}^T \\ &= \left(\mathbf{a} d^{1/2}\right)\left(\mathbf{h} d^{1/2}\right)^T = \mathbf{b}\mathbf{l}^T \end{aligned} \tag{4}$$

where $\mathbf{b}$ and $\mathbf{l}$ are the borrowing and the lending score vectors respectively.

Thus, we can transform the optimization problem (3) to be an equivalent problem of NMF:

$$\min_{\mathbf{b}\in\mathbb{R}_+^{n\times 1}\mathbf{l}\in\mathbb{R}_+^{n\times 1},} \frac{1}{2} \| \mathbf{W} - \mathbf{b}\mathbf{l}^T \|_F^2 \tag{5}$$

The following lemma holds:

Lemma 1 *The pair of vectors* $\mathbf{b}$ *and* $\mathbf{l}$ *are local minimizers of the function* $\frac{1}{2} \| \mathbf{W} - \hat{\mathbf{W}} \|_F^2$ *being* $\mathbf{l}$ *and* $\mathbf{b}$ *the non-negative right eigenvectors of* $\mathbf{W}\mathbf{W}^T$ *and* $\mathbf{W}^T\mathbf{W}$ *respectively.*

Proof. We permute matrix $\mathbf{W}$ in a way such that vectors $\mathbf{b}$ and $\mathbf{l}$ are partitioned as $(\mathbf{b}_+, \mathbf{0})^T$ and $(\mathbf{l}_+, \mathbf{0})^T$ with $\mathbf{b}_+,\mathbf{l}_+ > 0$. As a consequence $\mathbf{W}$ becomes

$$\mathbf{W} = \begin{pmatrix} \mathbf{W}_{11} & \mathbf{W}_{12} \\ \mathbf{W}_{21} & \mathbf{W}_{22} \end{pmatrix}$$

The Karush-Kuhn-Tucker conditions for the nonnegative matrix factorization problem can be rewritten as

$$\begin{aligned} &\begin{pmatrix} \mathbf{b}_+\mathbf{l}_+^T & \mathbf{0} \\ \mathbf{0} & \mathbf{0} \end{pmatrix}\begin{pmatrix} \mathbf{l}_+ \\ \mathbf{0} \end{pmatrix} + \\ &-\begin{pmatrix} \mathbf{W}_{11} & \mathbf{W}_{12} \\ \mathbf{W}_{21} & \mathbf{W}_{22} \end{pmatrix}\begin{pmatrix} \mathbf{l}_+ \\ \mathbf{0} \end{pmatrix} \geq \mathbf{0} \end{aligned}$$

and

$$\begin{aligned} &\begin{pmatrix} \mathbf{l}_+\mathbf{b}_+^T & \mathbf{0} \\ \mathbf{0} & \mathbf{0} \end{pmatrix}\begin{pmatrix} \mathbf{b}_+ \\ \mathbf{0} \end{pmatrix} + \\ &-\begin{pmatrix} \mathbf{W}_{11}^T & \mathbf{W}_{21}^T \\ \mathbf{W}_{12}^T & \mathbf{W}_{22}^T \end{pmatrix}\begin{pmatrix} \mathbf{b}_+ \\ \mathbf{0} \end{pmatrix} \geq \mathbf{0} \end{aligned}$$

This implies that $\mathbf{W}_{21}\mathbf{l}_+ \leq 0$ and $\mathbf{W}_{12}^T\mathbf{b}_+ \leq 0$. Since $\mathbf{W}_{21}, \mathbf{W}_{12}^T \geq 0$ and $\mathbf{b}_+,\mathbf{l}_+ > 0$ we can conclude that $\mathbf{W}_{21} = 0$ and $\mathbf{W}_{12}^T = 0$. Then from equation the complementarity constraints we have

$$\begin{aligned} \mathbf{b}_+ \circledast \left(\|\mathbf{l}_+\|_2^2\, \mathbf{b}_+ - \mathbf{W}_{11}\mathbf{l}_+\right) &= 0, \\ \mathbf{l}_+ \circledast \left(\|\mathbf{b}_+\|_2^2\, \mathbf{l}_+ - \mathbf{W}_{11}^T\mathbf{b}_+\right) &= 0 \end{aligned}$$

Since $\mathbf{b}_+,\mathbf{l}_+ > 0$ we have

$$\begin{aligned} \|\mathbf{l}_+\|_2^2\, \mathbf{b}_+ &= \mathbf{W}_{11}\mathbf{l}_+, \\ \|\mathbf{b}_+\|_2^2\, \mathbf{l}_+ &= \mathbf{W}_{11}^T\mathbf{b}_+ \end{aligned}$$

or, equivalently

$$\|\mathbf{b}_+\|_2^2 \|\mathbf{l}_+\|_2^2 \mathbf{b}_+ = \mathbf{W}_{11}\mathbf{W}_{11}^T\mathbf{b}_+,$$
$$\|\mathbf{b}_+\|_2^2 \|\mathbf{l}_+\|_2^2 \mathbf{l}_+ = \mathbf{W}_{11}^T\mathbf{W}_{11}\mathbf{l}_+$$

This means that the pair $(\mathbf{b},\mathbf{l})$ is a local minimizer of (5) if and only if $\mathbf{b},\mathbf{l}$ are non-negative eigenvectors of $\mathbf{W}\mathbf{W}^T$ and $\mathbf{W}^T\mathbf{W}$ respectively of the singular value $d = \|\mathbf{b}_+\|_2 \|\mathbf{l}_+\|_2$.

■

Therefore we can retrieve the original vectors $\mathbf{a}$ and $\mathbf{h}$ from (4), using the fact that the singular values of $\mathbf{W}$ are the square root of the eigenvalues of $\mathbf{W}\mathbf{W}^T$ and $\mathbf{W}^T\mathbf{W}$

$$\mathbf{a}=\frac{\mathbf{b}}{\sqrt{\|\mathbf{b}_+\|_2 \|\mathbf{l}_+\|_2}},$$
$$\mathbf{h}=\frac{\mathbf{l}}{\sqrt{\|\mathbf{b}_+\|_2 \|\mathbf{l}_+\|_2}}$$

In so doing we established a relationship between the HITS algorithm and the non-negative matrix factorization outcome for the rank-1 matrix case, namely the rank-one NMF solution is always a rescaled version of authority and hub scores obtained with the HITS algorithm.

Goodness of fit and community structure

In the main text we proposed a rule-of-thumb based on model fit to detect the number of communities in which the network can be partitioned into. To do so, we adopted a heuristic approach fixing at 90% the data variability we want to replicate, and looking for the number of communities that can jointly meet this goodness of fit. We also compute the number of communities able to explain the 95% and the 99% of the data variability. While in the first case the number of communities ranges from 5 to 25, in the second case the number of communities reaches 47. We choose the more conservative approach because using a high number of components faces the risk of overfitting noises.

Despite the fact that we are aware of the limitation of this proposal, the systematic investigation of the exact number of communities during different time is behind the scope of the paper. Nevertheless this section aims at shed some light about whether the negative correlation between the model fit computed with a rank-1 NMF ($K = 1$) and the traded volume is given by an increasing number of communities composing the network or if it is due to spurious factors.

Indeed, as long as the traded volume increases, the rank-1 representation of $\mathbf{W}$, that is $\hat{\mathbf{W}} = \mathbf{b}\mathbf{l}$ can result in a dense matrix because of some very high value in $\mathbf{b}$ or in $\mathbf{l}$ producing in this way an artificial low fit. To rule out this possibility we use the null model of Bargigli and Gallegati [2], [3] to create a randomized versions of the original network preserving, at the same time, the local topological properties.

In this way we obtain a network ensemble with the same sparsity of the original network but with random connection that we can employ as benchmark. We perform the same rank-1 decomposition for each of the randomized networks and then we take the mean of the fit. S1 Fig shows, for each period, the fit associated to the original data and the mean fit produced by the decompositions of the randomized networks. While the original data-set shows a “V” shaped model fit, the fit produced by the null model seems not to be affected by the traded volume changes over time having always values

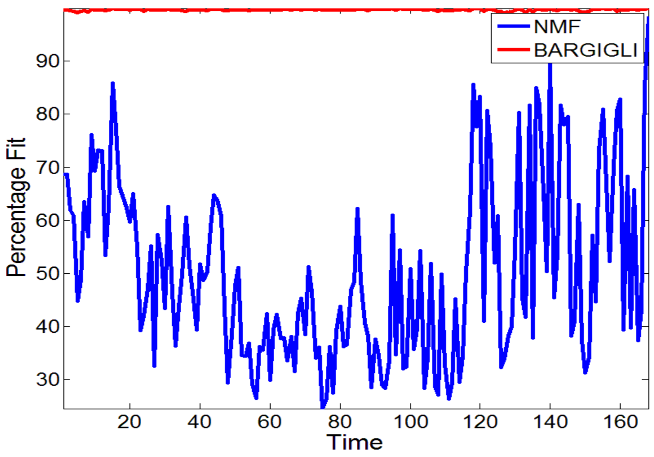

S1 Fig. Model fit comparison. Model fit comparison for rank-1 approximation of the original network (blue line) and for the network sample created by the null model (red line). While the original dataset shows a “V” shaped model fit, the fit produced by the null model seems not to be affected by the traded volume changes over time.

near 98%. This evidence reinforces the idea of a community structure in the e-MID interbank market that changes through time, depending on the transactions executed by the various entities in different periods.

Descriptive statistics for borrowing and lending scores

The dynamics of the borrowing and lending scores presented in the main text is complemented by the following statistics which provide a further insight about the evolution of lending and borrowing activity of the institutions over time.

The upper panels of S2 Fig show, for each period, the mean value (blue) of the borrowing (a) and lending (b) scores and the corresponding standard deviation (red); the bottom panels display the same statistics computed for each bank. In order to generate these statistics we have first averaged the scores of each bank across the communities it belongs to. In such a way we obtain an average systemic importance related to each bank. The top left panel of S2 Fig presents the most stable statistics. In fact, along time, the mean values of the borrowing scores averaged across banks, and the relative standard deviation, behave approximately in the same way for the whole sample. This is no longer true for the lending scores (top right panel of S2 Fig) where the standard deviation displays peaks approximately in the years 2000, 2009 and 2012. Comparing the upper and the lower panels it is easy to note how the statistics computed over time (top panels) are more stable then the ones computed over banks (bottom panels). Indeed, in the bottom panels of S2 Fig, the standard deviation follows the same path of the mean values of the statistics. This means that over time each bank behaves approximately at the same manner or, in other words, banks with a high systemic importance remain central for all the periods.

Together with the descriptive statistics, our results can also be interpreted looking at the following tables, in which we highlight the ranking of the borrowing and lending scores across time and across banks.

S1 Table gives the first ten periods associated with the highest borrowing and

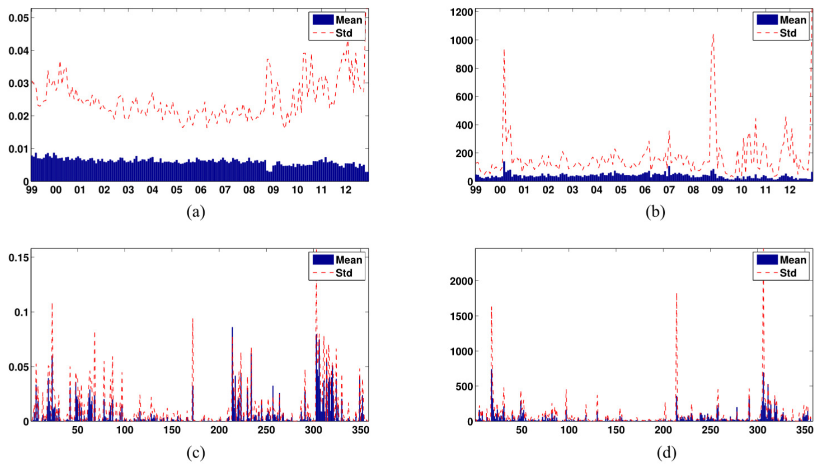

S2 Fig. Summary statistics of the borrowing/lending scores. Panel (a) shows the statistics computed over time for the borrowing score, showing in each period what is the mean value of the borrowing score and its standard deviation. Panel (b) shows the same statistics for the lending scores. The bottom subplots show the statistics computed over the number of banks. Panel (c) shows, for each bank the mean value of the borrowing score and its standard deviation. Panel (d) encompasses the same statistics for the lending score.

S1 Table. Top 10 periods and banks with the highest borrowing and lending scores.

Periods	Max B	Periods	Max L	Banks	Max B	Banks	Max L
12-31-12	1,000	12-31-12	23166,0	IT0253	1,000	IT0159	23166,0
11-30-12	1,000	11-21-08	17979,8	GB0023	0,667	IT0256	17979,8
05-26-10	0,667	10-22-08	16813,7	DE0012	0,664	DE0006	8094,1
06-30-12	0,667	03-04-00	16527,2	IT0111	0,500	IT0019	4687,5
04-26-10	0,667	12-22-08	9898,0	IT0267	0,500	DE0008	4637,7
12-29-11	0,664	08-26-10	8094,1	IE0003	0,500	IT0255	4129,4
02-28-12	0,662	11-28-11	8008,8	IT0261	0,433	IT0143	3447,7
08-26-10	0,661	05-26-10	6333,4	IT0260	0,429	IT0237	3412,3
03-04-00	0,500	06-04-00	6168,3	IT0256	0,428	IT0261	3211,5
07-30-12	0,500	04-26-10	5667,9	IT0007	0,389	DE0021	3058,1

S2 Table. Top 10 periods and banks with the highest borrowing and lending scores mean.

Periods	Mean B	Periods	Mean L	Banks	Mean B	Banks	Mean L
12-03-99	0,009	03-04-00	139,4	IT0159	0,086	DE0006	737,9
03-03-99	0,009	01-18-07	106,8	IT0253	0,079	IT0256	694,1
09-02-99	0,009	11-21-08	84,8	IT0256	0,074	IT0261	526,4
08-03-99	0,008	06-04-00	79,8	IT0180	0,062	IT0159	358,5
01-03-00	0,008	05-04-00	75,4	DE0012	0,060	DE0008	325,0
01-01-99	0,008	10-22-08	75,1	IT0265	0,056	IT0237	317,1
05-12-03	0,008	10-13-04	73,1	IT0271	0,051	IT0269	309,6
10-03-99	0,008	03-18-06	71,8	IT0254	0,047	FR0008	291,4
12-05-00	0,008	11-17-06	70,2	IT0168	0,044	IT0255	282,8
01-31-99	0,007	12-31-12	64,7	IT0162	0,041	IT0258	260,6

S3 Table. Top 10 periods and banks with the highest borrowing and lending scores standard deviation.

Periods	Std B	Periods	Std L	Banks	Std B	Banks	Std L
12-31-12	0,053	12-31-12	1222,6	IT0253	0,158	IT0256	2459,6
11-30-12	0,053	11-21-08	1040,9	DE0012	0,109	IT0159	1831,3
02-28-12	0,044	03-04-00	938,9	IT0111	0,094	DE0006	1628,9
12-29-11	0,039	10-22-08	919,6	GB0023	0,083	DE0008	572,7
06-30-12	0,039	12-22-08	560,8	IT0256	0,080	IT0261	517,7
05-26-10	0,039	11-28-11	455,4	IT0159	0,078	DE0021	479,1
08-26-10	0,039	08-26-10	444,1	IT0261	0,078	IT0237	462,1
04-26-10	0,039	04-06-00	395,1	IT0267	0,070	IT0019	453,3
11-21-08	0,037	02-28-12	371,8	IT0180	0,068	IT0204	452,0
10-22-08	0,037	01-18-07	356,0	IT0265	0,067	FR0008	441,5

lending scores, confirming the fact that institutions displayed a high level of systemic importance during the years of the financial crisis.

On the other hand, S1 Table also emphasizes how especially Italian banks (but also some German and British banks) have the highest level of borrowing and lending scores, confirming the fact that, while during the pre-crisis period foreign financial institutions were the most systemically important lenders, when the crisis unfolded foreign banks suddenly stopped to lend to other institutions and Italian banks increased their lending activities.

S2 Table reports the first ten periods associated with the highest average borrowing and lending scores together with the top 10 banks. It is worth to note that these periods mostly correspond to the first part of the sample (1999-2000), in which the lending activity was almost stable, as also S2 Fig suggests. Again, we can also observe that the majority are Italian banks (and some German and French banks) confirming the role such institutions exerted as main lenders and borrowers.

S3 Table displays the first ten periods associated with the highest standard deviations for borrowing and lending scores together with the top 10 banks. As opposed to the previous table, the ten periods in which the borrowing and lending scores exhibit the highest variability are associated with the recent financial crisis and the years just after that. Additionally, the banks with the highest standard deviation in the borrowing and lending scores are still related to Italian (and some German and French) institutions, and this is associated to the course of the economic trend that characterized their borrowing and lending activity during the whole sample.

Finally, in order to prove the convergence of our technique we record for each time period the relative error made at each iteration. S3 Fig in S1 File shows how the relative error made by our approximation $\mathbf{W} \equiv \mathbf{BL}$ decreases as long as the number of iteration increases and approaches 0.1 (10%) after 100 iterations. This happens for all the 168 time periods under the analysis.

The E-mid interbank market dataset

The rapid response to financial shocks in the interbank market, such as decreased liquidity and higher interest rate, increased the focus and the attention in the interbank market. Within this market, the overnight segment is the one where banks' primary focus is to meet their short-term liquidity needs and to ensure that the trading day is closed with a balanced position. In the overnight money market, reserve requirements and uncontrolled factors, such as monetary policy or liquidity shocks, are the two main ingredients governing the majority of all transactions between financial institutions.

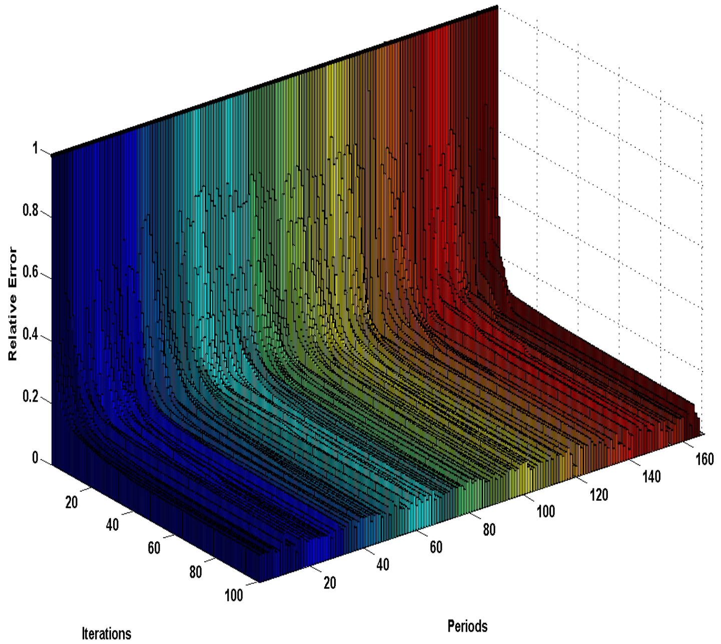

S3 Fig. Model convergence. Relative error as a function of the number of iteration for all the time periods under analysis.

When there is a gap between the reserve needs and what can be met through the refinancing operations, financial companies explore unsecured market with the aim of find shortest-maturity liquidity. The only official source of data about overnight market is the euro overnight index average (EONIA) published by the European Banking Federation (EBF). However, the lack of data in the EONIA make it impossible to examine the market in detail.

Therefore the e-MID turns out to be the only electronic platform where a subset of transactions in the overnight segment is captured. The e-MID is an electronic market based in Milan that was developed for uncollateralized interbank transactions in Italian Lira in 1990 and re-denominated into Euro in 1999. The e-MID market is highly liquid, it is continuously open between 8am - 6pm CET and it is available for interbank transactions to any bank operating in the European interbank market. Contracts are settled at different maturities ranging from overnight up to one year, with the largest bulk of the transactions settled overnight.

Its main difference from secured markets is that participants can actively choose each other. There are two type of participants in the e-MID transactions: quoter and aggressor. A credit institution that places its order first with a proposal to lend or borrow is called the quoter, and those who can pick a quote and exercise a proposal is called the aggressor. Once an aggressor bank responds to a quote, a negotiation between the two parties begins, whereby each party is able to modify the conditions until both parties agree on the deal or until one of them rejects it. As an electronic platform, the e-MID's overnight segment accounts for more than 90% of all transactions in the market. In the present paper, we use the monthly aggregation of the daily overnight transactions from the e-MID electronic platform for the purposes of this study.

Our dataset includes tick-by-tick data of the e-MID from January 1999 to December 2012. We have detailed information about each transaction; time, volume of trade,

maturity, interest rate, the side of the transaction (buy/sell) and the code of the banks acting as quoter and aggressor, country of origin and size of both parties. The interest rate is expressed as annual rate and the volume of the transaction is provided in millions of Euros. The e-MID market includes contracts with maturities varying from one day to one year. We restrict our analysis to overnight (O/N) and the overnight long (ONL), which consists of more than 90% of all e-MID transactions as the interbank market is mainly a market for short-term trades.

S4 Table. Descriptive statistics

Descriptive Statistics

	Num. trans.	% trans. IT. B.	% trans. IT. L.	Volume (EUR bn)	% Vol. IT. B.	% Vol. IT. L.	Av. daily int. rate (% p.a.)	Av std. int. rate
1999	169,889	99.87	99.99	2,493.39	99.64	99.97	2.73	0.03
2000	149,892	99.24	99.22	3,037.63	97.59	94.99	4.12	0.03
2001	130,216	97.10	98.18	3,058.66	83.65	87.11	4.38	0.04
2002	135,996	94.45	98.12	3,574.44	73.63	85.99	3.27	0.02
2003	120,271	93.08	95.48	3,900.72	65.26	74.79	2.31	0.03
2004	113,503	91.41	91.42	4,829.25	58.78	60.91	2.05	0.02
2005	108,727	89.48	89.08	4,962.35	55.68	56.03	2.08	0.02
2006	103,292	88.52	86.64	5,456.94	49.76	49.31	2.83	0.02
2007	99,029	88.23	86.39	5,248.56	44.84	44.90	3.87	0.05
2008	82,693	93.12	91.93	3,021.92	62.35	58.20	3.82	0.08
2009	53,991	97.64	97.42	1,246.62	77.78	75.59	0.63	0.08
2010	50,180	98.97	97.70	1,231.89	83.86	75.01	0.43	0.06
2011	47,070	99.11	98.62	1,115.95	85.73	81.70	1.04	0.15
2012	28,509	99.58	99.49	640.35	93.94	92.50	0.21	0.04

Note: The columns % trans. IT. B. and % trans. IT. L. correspond to the percentage of all transactions in which the borrowing/lending bank was Italian/Foreign. Similarly, the columns % Vol. IT. B. and % Vol. IT. L. correspond to the percentage of the total traded volume that was borrowing/lent by Italian/Foreign banks.

S4 Table shows the descriptive statistics of the E-mid interbank market indicating a systematic reduction of the number of transactions over time. Columns 2 and 3 display the percentage of all the transactions in which the borrower/lender was an Italian bank. It has to be noticed that Italian banks execute the biggest number of transactions and this trend has little variation over the sample period. On the contrary the yearly transaction volumes show a very different behavior than the number of transactions. The traded volume increases steadily until the year 2007 and declines sharply thereafter. Columns 5 and 6 report the share of the traded volume borrowed/lent by Italian banks emphasizing that Italian banks are no longer the most important borrowers/lenders over the entire sample period. Foreign banks, although posting a smaller amount of transactions, show significantly larger volumes than those of Italian banks, particularly in the middle of the sample. Finally, the table shows that interest rates closely follow the EONIA rates (not shown in the table) and that the dispersion of the interest rate increases again since 2007.

S4 Fig shows the time evolution of the number of transactions (left) and the traded volume (right) from 1999 to 2012 at a monthly frequency, As before we make again a distinction between the behavior of Italian and foreign borrowers (up) and lenders (bottom) respectively. As reported in the table, also the figure emphasizes the

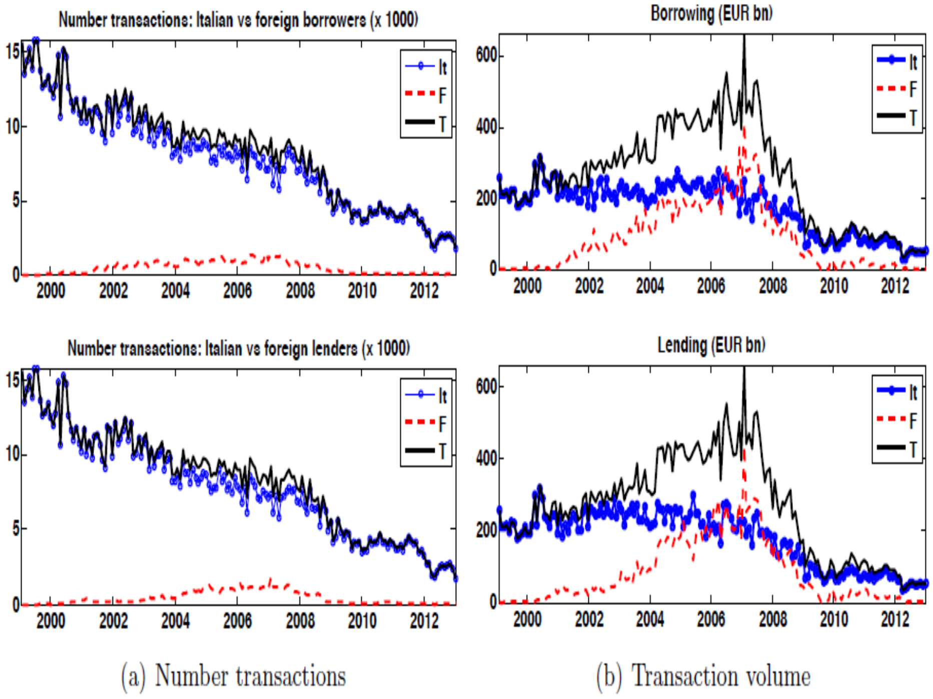

Figure D. Italian vs Foreign Banks dynamics.

continuous and linear decline in the number of transaction, which is mostly explained by the conduct of Italian banks. Foreign banks increased their participation until 2007 and quickly reduced it afterwards.

Furthermore the dynamics of the traded volume is very different. The figure shows that the transactions volume smoothly increases until 2007, and this behavior is mostly explained by the orders posted by foreign rather than Italian banks. Since the beginning of the financial crisis, foreign banks quickly retreated from the market, which lead to a sharp decline in the overall trading volume. Moreover the dispersion of foreign banks' borrowing and lending behavior was in general more volatile than that of Italian banks. The volatility of foreign banks behavior increased until the beginning of the financial crisis, and declined thereafter together with their overall trading volumes.

References

1. Kleinberg, J.M. Authoritative Sources in a Hyperlinked Environment. *J ACM* 46, 604-632 (1999).

2. Bargigli, L., & Gallegati, M. Finding communities in credit networks. *Economics*, 7(17), 1 (2013).

3. Bargigli, L., & Gallegati, M. Random digraphs with given expected degree sequences: a model for economic networks. *Journal of Economic Behavior & Organization*, 78(3), 396-411 (2011).
