# Supplementary figures and images for: Discovering SIFIs in Interbank Communities
Source: PLoS One. 2016 Dec 21;11(12):e0167781. doi: 10.1371/journal.pone.0167781 (PMC5176285; doi:10.1371/journal.pone.0167781)

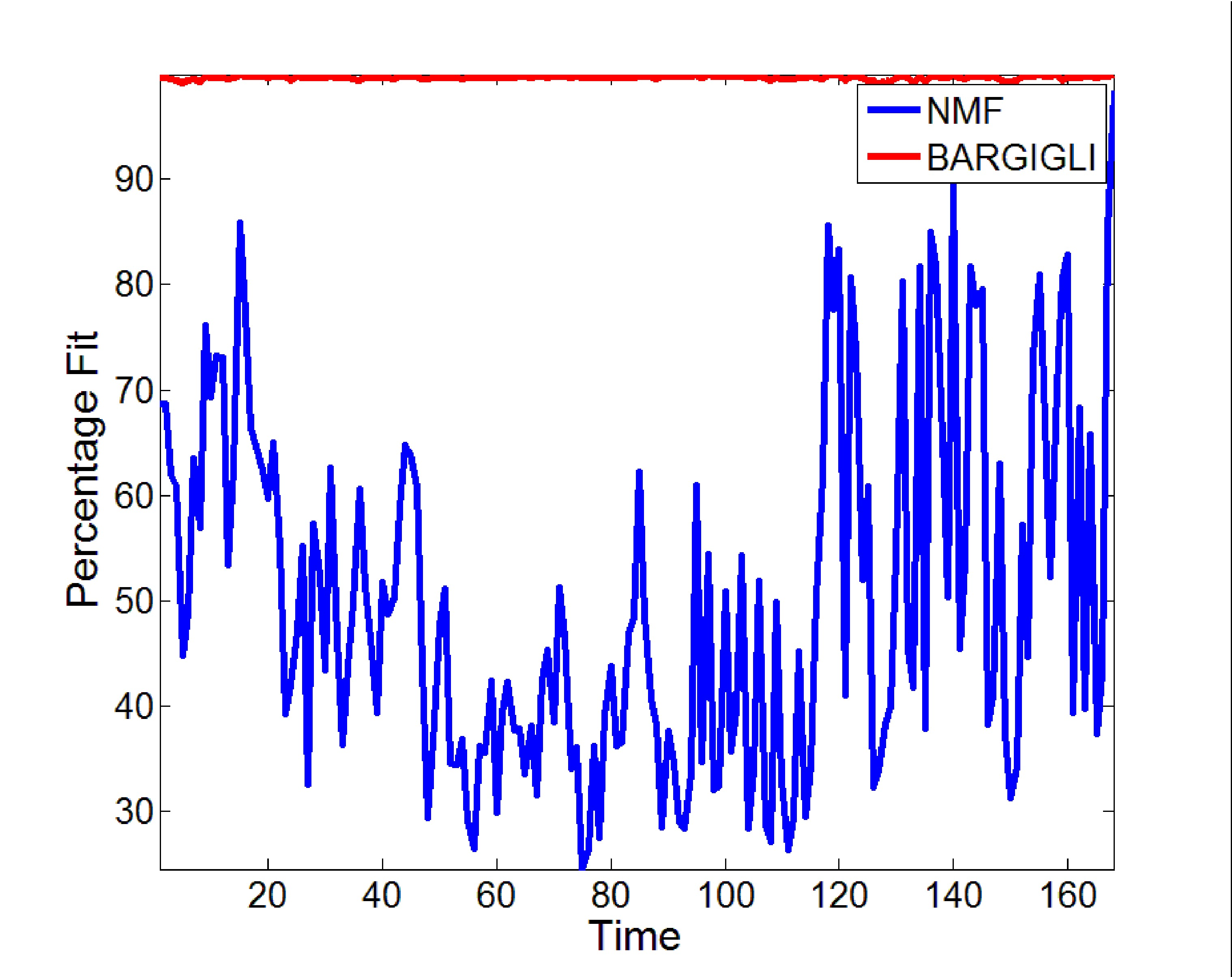

Supplement: S1 Fig — Model fit comparison for rank-1 approximation of the original network (blue line) and for the network sample created by the null model (red line). While the original dataset shows a “V” shaped model fit, the fit produced by the null model seems not to be affected by the traded volume changes over time. (TIF) [file pone.0167781.s002.tif]

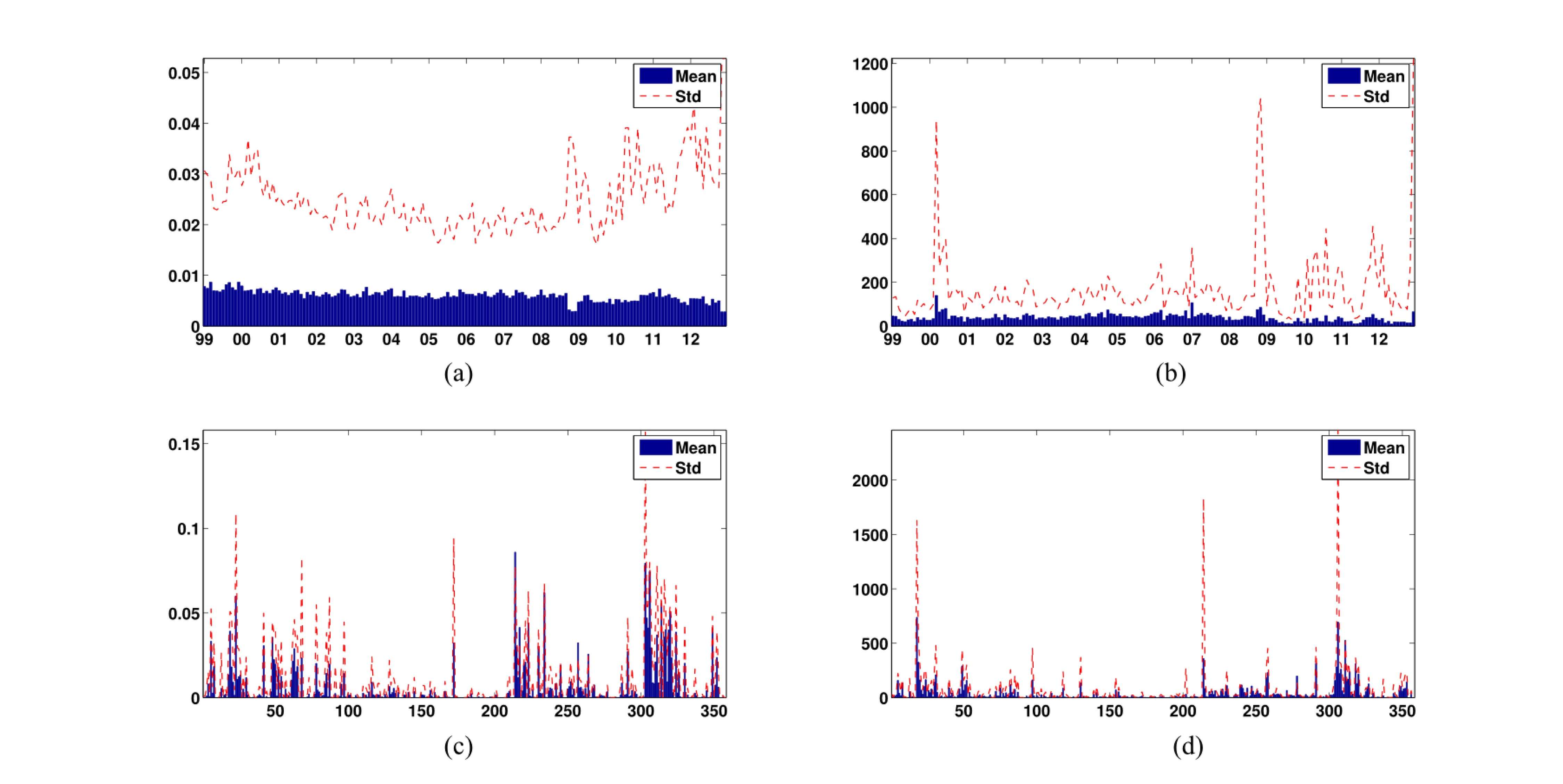

Supplement: S2 Fig — Panel (a) shows the statistics computed over time for the borrowing score, showing in each period what is the mean value of the borrowing score and its standard deviation. Panel (b) shows the same statistics for the lending scores. The bottom subplots show the statistics computed over the number of banks. Panel (c) shows, for each bank the mean value of the borrowing score and its standard deviation. Panel (d) encompasses the same statistics for the lending score. (TIF) [file pone.0167781.s003.tif]

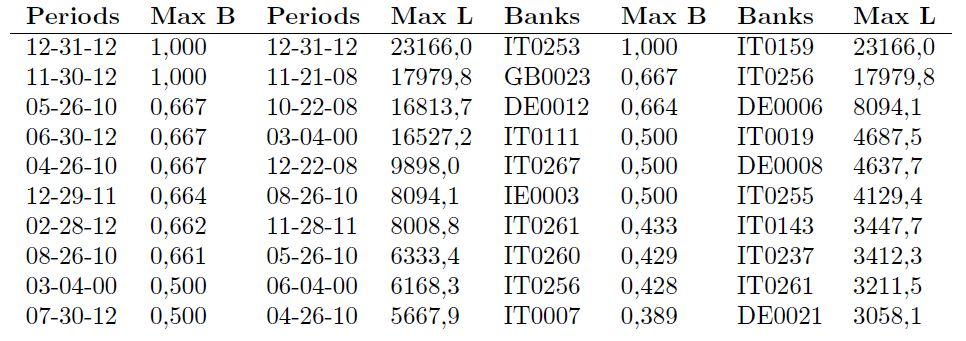

Supplement: S1 Table — (TIF) [file pone.0167781.s004.tif]

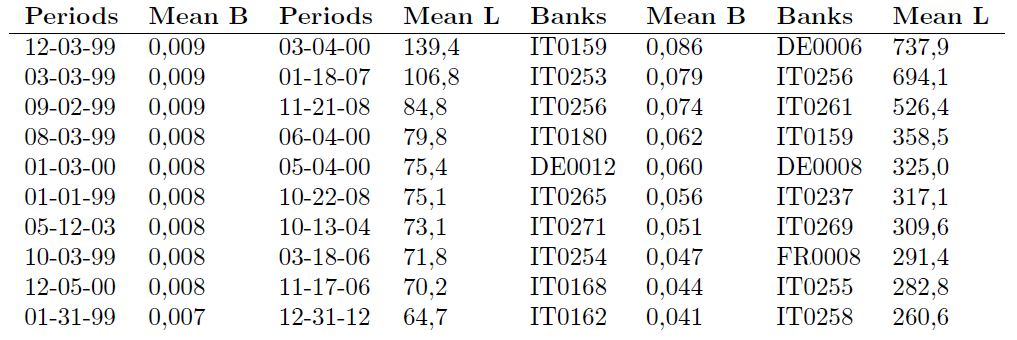

Supplement: S2 Table — (TIF) [file pone.0167781.s005.tif]

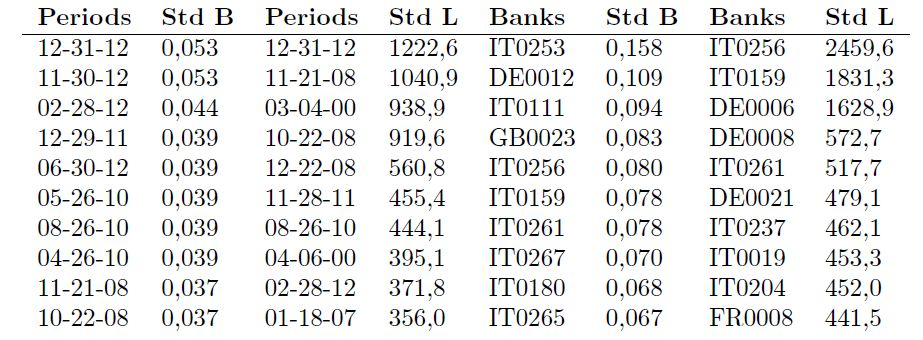

Supplement: S3 Table — (TIF) [file pone.0167781.s006.tif]

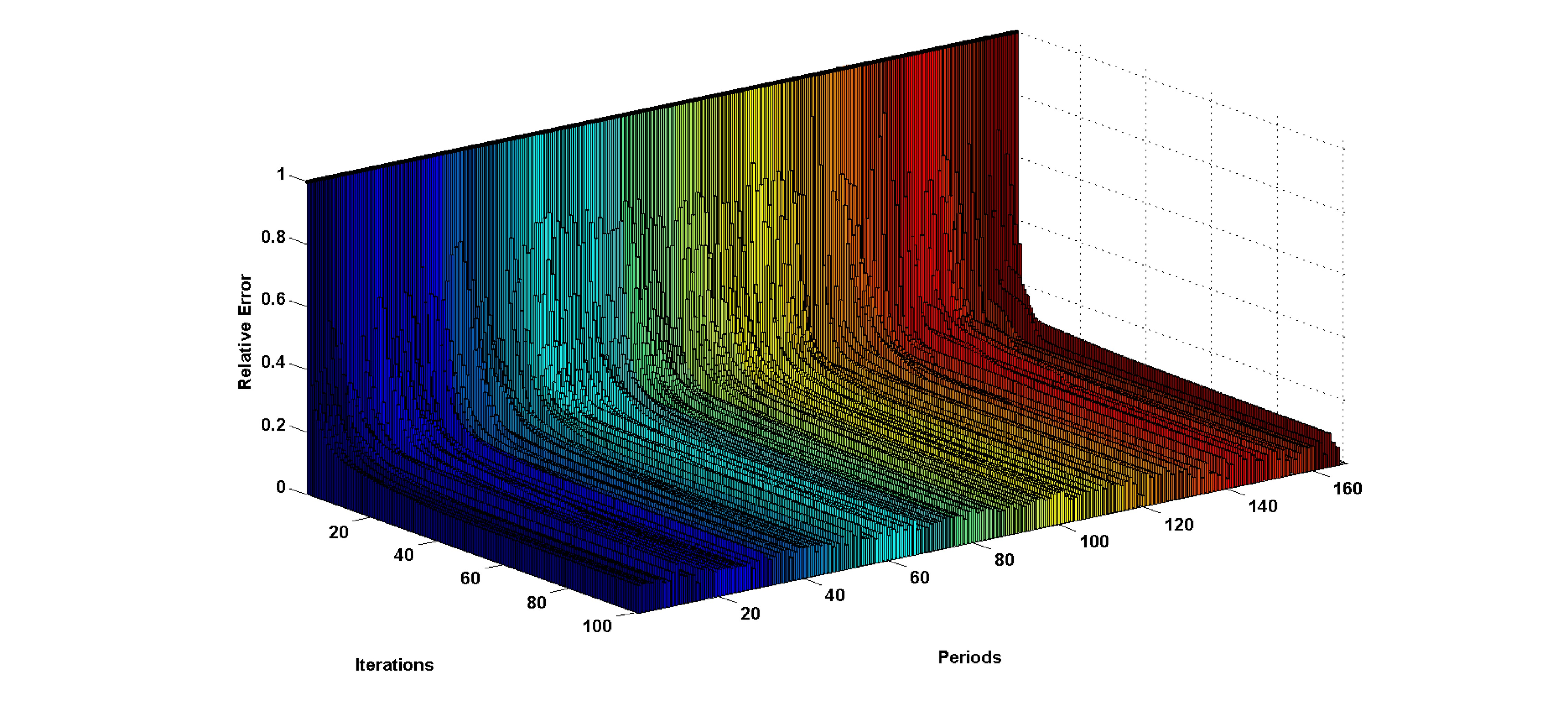

Supplement: S3 Fig — Relative error as a function of the number of iteration for all the time periods under analysis. (TIF) [file pone.0167781.s007.tif]

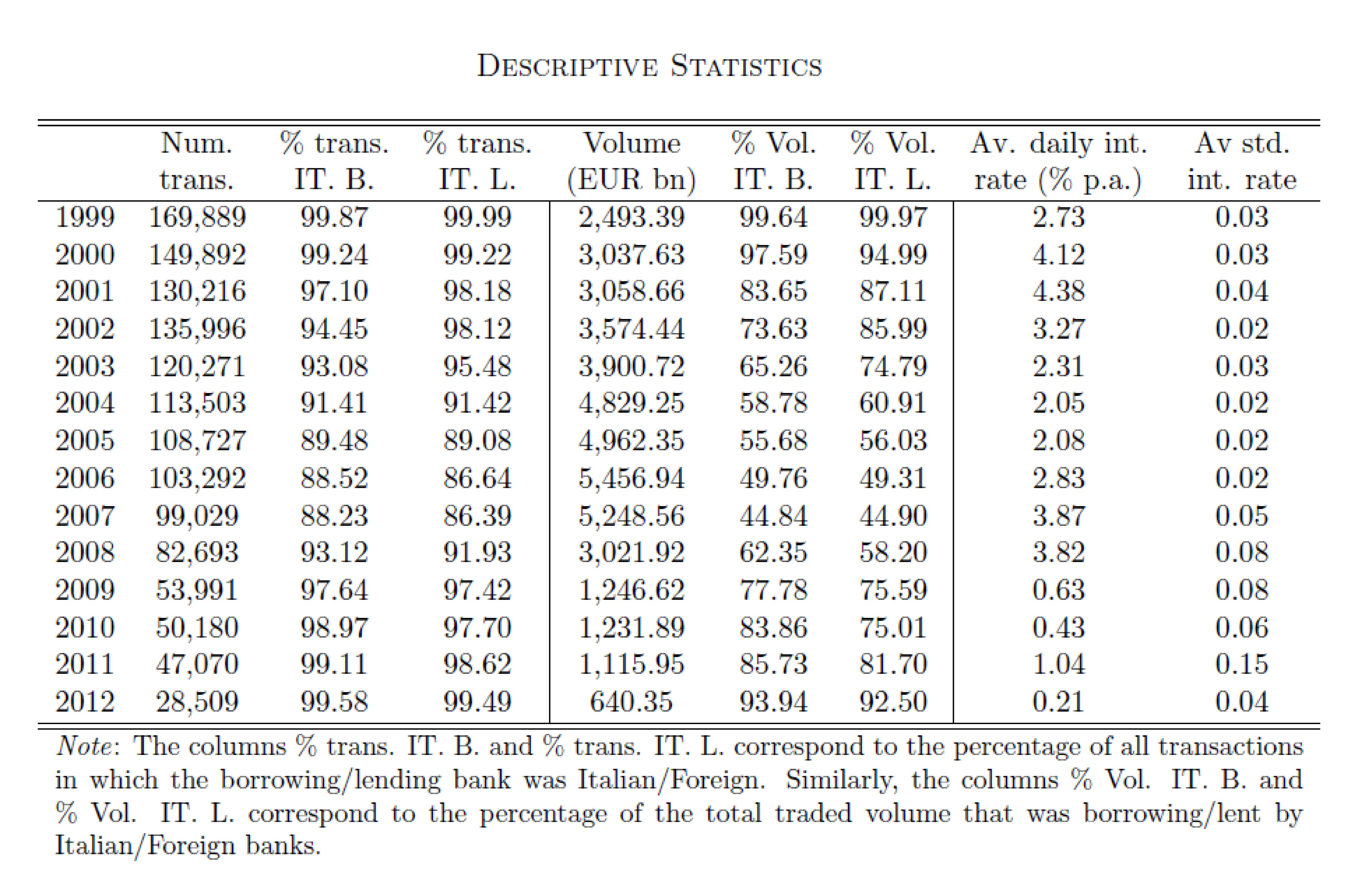

Supplement: S4 Table — (TIF) [file pone.0167781.s008.tif]

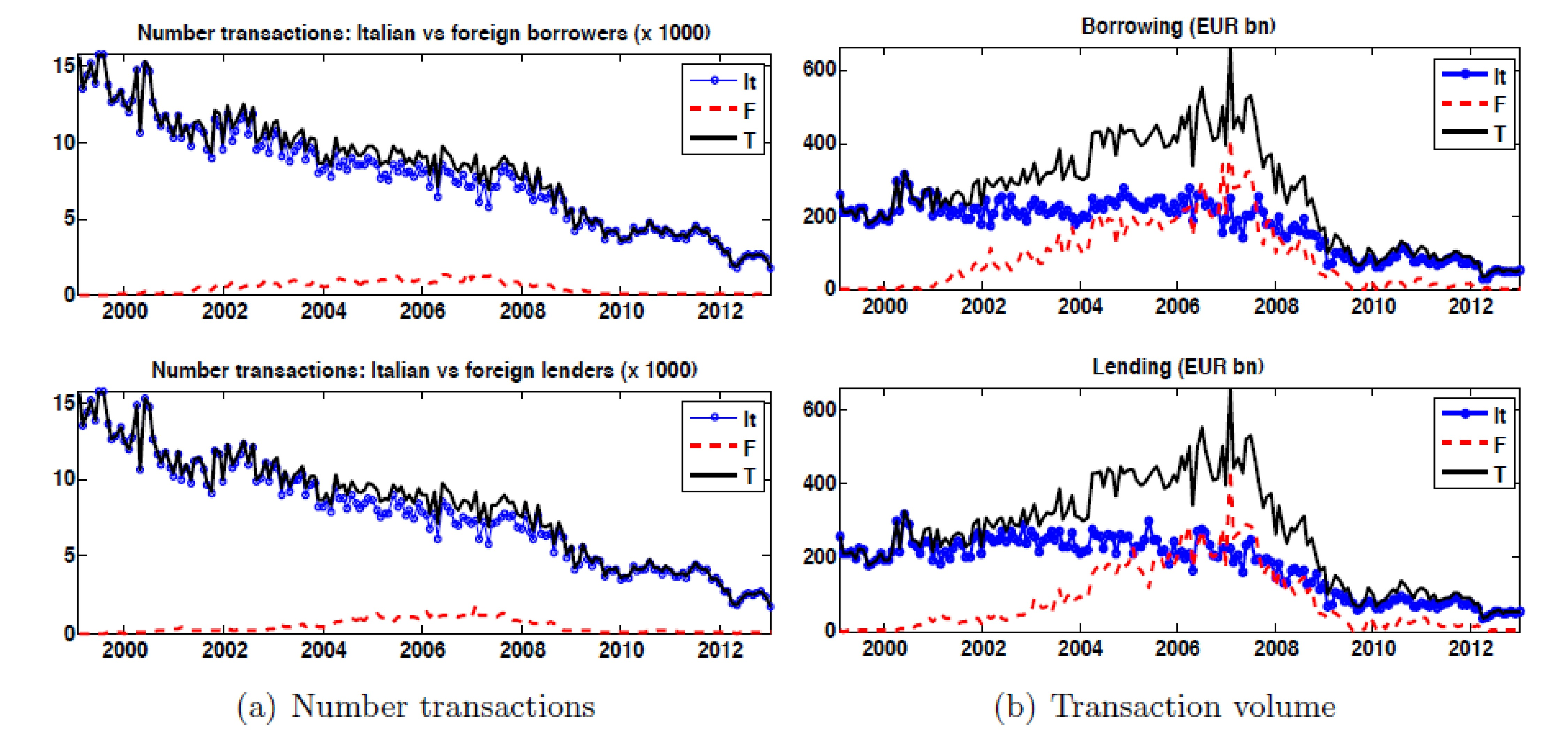

Supplement: S4 Fig — (TIF) [file pone.0167781.s009.tif]
